# Inflammatory signaling mechanisms in bipolar disorder

**DOI:** 10.1186/s12929-021-00742-6

**Published:** 2021-06-11

**Authors:** Gregory H. Jones, Courtney M. Vecera, Omar F. Pinjari, Rodrigo Machado-Vieira

**Affiliations:** Department of Psychiatry and Behavioral Sciences, University of Texas Health Science Center at Houston (UTHealth), 1941 East Road, Houston, TX 77054 USA

**Keywords:** Bipolar, Inflammation, Purine, P2X7, Mitochondria, BDNF, Glutamate, NLRP3

## Abstract

Bipolar disorder is a decidedly heterogeneous and multifactorial disease, with a high individual and societal burden. While not all patients display overt markers of elevated inflammation, significant evidence suggests that aberrant immune signaling contributes to all stages of the disease, and likely explains the elevated rates of comorbid inflammatory illnesses seen in this population. While individual systems have been intensely studied and targeted, a relative paucity of attention has been given to the interconnecting role of inflammatory signals therein. This review presents an updated overview of some of the most prominent pathophysiologic mechanisms in bipolar disorder, from mitochondrial, endoplasmic reticular, and calcium homeostasis, to purinergic, kynurenic, and hormonal/neurotransmitter signaling, showing inflammation to act as a powerful nexus between these systems. Several areas with a high degree of mechanistic convergence within this paradigm are highlighted to present promising future targets for therapeutic development and screening.

## Background

Bipolar disorder (BD) is a highly complex, multifactorial disease with significant clinical heterogeneity, pleiotropy, and pathophysiological basis. Subjects with BD display high rates of inflammatory comorbidities [1, 2]. This review aims to provide a convergent overview of biological systems and pathways (purinergic, mitochondrial, inflammatory, immune, kynurenic, and hormonal) having inflammation as the key mediator. Specifically, we aim to show how dysfunctional mitochondrial respiration can compound oxidative stress and increase inflammation and how proteins which mediate mitochondrial and endoplasmic reticulum communication link calcium-based signaling to immune cell activation. We further describe how purine metabolites can modulate of mood symptoms through cytokine signaling, and how chronic stress-hormonal overactivation can exacerbate disease through inflammatory pathways. Special attention is given to purines, neuroplasticity, mitochondrial/cellular damage signals, and the nod-like receptor family pyrin domain-containing 3 (NLRP3) inflammasome. These processes appear to exert pervasive, mutualistic influence across a wide range of cell types and systems, merging seemingly disparate pathologic signals via systemic and local inflammation.

Given the frequency of immune-inflammatory comorbidities in BD, we suggest that a detailed understanding of this dynamic may yield significant benefit in treating not only mood symptoms, but many other disorders associated with BD. In presenting several convergent mechanisms, we suggest that future therapeutic efforts in these systems connected to inflammatory signaling may hold particular relevance in screening and drug development.

## Intracellular signaling and inflammation

Epidemiological evidence shows BD is associated with increased rates of inflammatory comorbidities including numerous autoimmune conditions, hypersensitivity reactions (asthma, seasonal allergies), and cardiometabolic diseases [2]. Though causality in this regard has yet to be established, recent research indicates that the relationship is likely bidirectional [2, 3]. Patients with BD demonstrate both central and peripheral elevations in proinflammatory elements (cytokines, chemokines, prostaglandins, acute-phase reactants, oxidative/nitrosive species), increased inflammatory gene expression, as well as aberrant cellular (T-cell, monocyte, microglial) and complement activation [2, 4, 5].

Despite some variation across studies, overall, patients with BD seem to consistently demonstrate higher serum concentrations of TNF-α, soluble TNF-receptor 1 (sTNF-R1), IL-1β, IL-4, soluble IL-2 receptor (sIL-2R), and soluble IL-6 receptor (sIL-6R) compared to healthy controls [4]. C-reactive protein (CRP), a nonspecific acute phase reactant synthesized in response to IL-1 and IL-6 secretion, has garnered mixed results in bipolar studies, but appears to be significantly associated with mania [6].

Specific cytokine expression patterns appear to be highly dependent on stage (early, late) and phase (manic, euthymic, depressed) of the disease [7], as well as medication status [8]. Interestingly, elevations in pro-inflammatory cytokines (especially IL-6) during depressive phases increase the likelihood of a subsequent transition to mania. Alterations in both IL-6 and TNF-α have also been associated with decreased BDNF expression in some studies, suggesting a cooccurrence of broader neuroplastic changes during these periods [5, 7, 9].

**Fig. 1:**
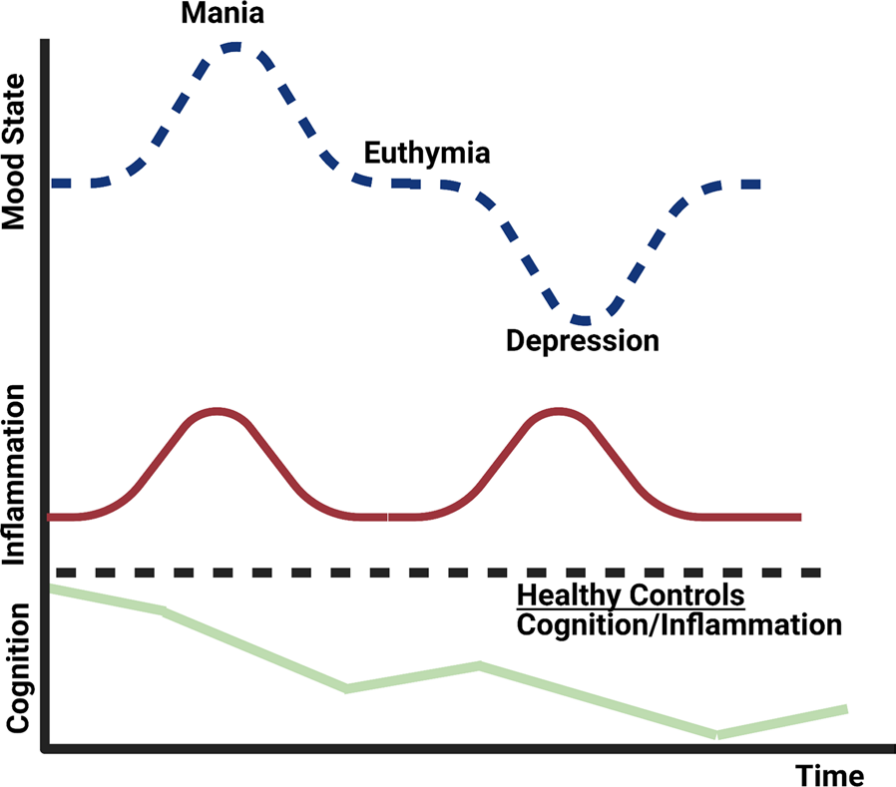
Inflammation and the neuroprogressive hypothesis. The graph above represents a theoretical model for clinical deterioration in BD. Of note, BD is a highly heterogeneous disease, which entails certain caveats to this model [12]. Notwithstanding, it appears that a significant portion of patients experience neuroprogression, that is, a progressive decline in neurocognitive function, associated with increasing frequency of mood (particularly manic) episodes. Notably, neurocognitive deficits (albeit less prominent) are evident, possibly as early as the prodromal phase [13]. Most patients experience a chronic, low-grade inflammatory state, which appears to be upregulated in concert with acute mood episodes. It is suggested that prolonged microglial activation compounds oxidative stresses, contributing to the progressive decline in neurocognitive function and neurostructural changes observed during the course of BD [12, 14]. Adapted with permission from Rosenblat and McIntyre, 2016 [15]

Overall, evidence suggests that many BD patients experience a chronic, low-grade inflammatory state that may be differentially enhanced during acute mood episodes, which may exacerbate other pathological mechanisms mentioned throughout this review and contribute to overall cognitive decline and neuroprogression [5, 10, 11] (see Fig. 1).

Notably, TNF-α seems to have special significance, appearing consistently elevated across all stages of mania and depression [4, 7]. During inflammatory reactions, TNF-α, secreted by microglia and other central nervous system (CNS) sources, plays a central role in altering BBB permeability and recruiting peripheral monocytes to CNS [5]. Upon entry, these cells then exponentiate cytokine release and sustain a pro-inflammatory milieu in the CNS, in part via upregulation of NLRP3 inflammasome activity (discussed in Sect. 4) [16]. This positive feedback loop is suggested to contribute to the chronic neurodegeneration observed in BD, in part by modulating GSK-3β activity—a key regulator of mitochondrial metabolism, DNA repair, inflammation, and apoptosis, which has been extensively implicated in BD [4].

Indeed, TNF-α has been shown to modulate many dysfunctional processes in BD including synaptic plasticity, neurotransmission, neurogenesis, neuronal survival, apoptosis, and even cognitive/behavioral functioning [4, 5, 17]. Immune system hyperactivation, propagated by increased serum TNF- α and IL-1 levels, correlate with increased risk of depression and cognitive impairment as well as decreased treatment responsivity, especially to lithium [18]. Additionally, peripheral sTNF-R1 levels positively correlate with disease severity, decreased cognitive function, and psychotic features in bipolar patients [17, 19].

Recent findings have also generated a growing interest in the role of the complement cascade in psychiatric disorders. Secreted by microglia, astrocytes, and leptomeningial cells, various complement proteins mediate chemoattraction during early neurogenesis, developmental synaptic pruning, neuronal survival, and immune surveillance [20–22]. Complement proteins also appear to exacerbate inflammation, predominately via NLRP3 activation (see Sect. 4 and Fig. 3).

In support of this model, Kucharska-Mazur et al. demonstrated increased peripheral concentrations of C3a and C5a in lithium-naïve patients suffering from BD for a minimum of one decade [23]. Other alterations in complement activity have also been demonstrated across several other studies. As with cytokines, the specific changes observed seem highly dependent on disease phase and medication status, especially with regards to lithium [21, 24]**.** Discrepancies notwithstanding, the major histocompatibility complex (MHC) region containing several complement genes (C2, C4, Factor B) has been associated with developing BD [25].

On a cellular level, bipolar patients have demonstrated increased T cell activation (higher Th1/Th2 ratio) and decreased in T regulatory cell activity. Patients also display signs of early T-cell senescence, which has been linked with early aging and neuroprogression [26]. T-cell alterations appear to be present in manic and depressed phases but tend to normalize after successful treatment/remission [4, 26].

Conversely, PET imaging and post-mortem tissue analysis suggest that microglial/monocyte overactivity persists in the hippocampus and prefrontal cortex regardless of disease status [27–29]. Increased monocyte inflammatory gene expression, activation, and migration have also been observed in the offspring of BD patients [30]. Moreover, adolescent offspring of bipolar patients appear to display subtle reductions in T regulatory cell populations, which are inversely correlated with inflammatory monocyte activity [31]. While the latter findings did not predict progression to psychopathology, this evidence would suggest at least some immune/inflammatory contribution to the characteristic heritability, accelerated aging, and neuroprogression seen in BD [4, 26].

Given the pervasive influence of immune/inflammatory dysfunction on BD and its numerous comorbidities, significant potential exists in targeting this system [32]. In terms of antidepressant efficacy for anti-inflammatory medicines, studies of individual agents have generated variable results, however recent meta-analyses suggest there may be a “class effect” overall, with a moderate to large effect size [33, 34]. Likewise, proof of concept studies for minocycline, CoQ10, and pioglitazone have generated moderate to large antidepressant effect sizes [32, 35, 36]. Anti-cytokine pharmacotherapies including TNF- α blockers (etanercept and infliximab) have been shown to attenuate depressive mood and inhibit cognitive decline in BD and Major Depressive Disorder (MDD) [5].

Fewer studies have assessed the anti-manic efficacy of this class, however preliminary results for adjunctive celecoxib and N-acetylcysteine (NAC) are promising [32]. Significant reductions in mania have also been demonstrated with the use of l‐tryptophan, magnesium, folic acid, and branched-chain amino acids (BCAAs) [37]. Omega-3 polyunsaturated fatty acids (omega-3s) have garnered mixed results for bipolar depression and negative evidence for mania. Limited evidence suggests no significant benefit for most anti-inflammatory medications as maintenance therapy [32]. Other inflammation-reducing interventions (probiotics, curcumin, colchicine, and ketogenic diets) have been proposed with some promising initial findings. However, they currently lack sufficient evidence to be properly evaluated for widespread use [32].

While some of these compounds may demonstrate efficacy as monotherapies, ultimately, this class may prove most effective as augmentation therapy for conventional BD treatments. Importantly, numerous studies have shown anti-inflammatory agents to be most beneficial in BD patients with elevated baseline inflammatory markers [35, 36, 38, 39]. As such, establishing standard panels for identification of this subgroup prior to initiating treatment, and tracking cytokine changes with patient outcomes should be prioritized in future trials.

Overall, it appears that inflammatory signaling mediates a strong connection between cellular stress, neuronal viability, and overt symptomatology. The hypothesis that BD incurs a low grade, proinflammatory state, exacerbated during acute mood episodes is potentially reinforced by the notion that anti-inflammatory therapies appear to be far more efficacious in mania and depression than in the maintenance phase.

Currently, there is no Food and Drug Administration-approved agent for the management of cognitive symptoms in BD. Though many of these trials would suggest a lack of conventionally defined efficacy as maintenance therapy, initial trials with anti-TNF medications have demonstrated the potential to attenuate cognitive decline in many patients. Going forward, it would be prudent to determine whether early interventions focused on reducing inflammation can replicate these findings, given the importance of cognitive performance in predicting functional impairment and overall wellbeing in BD [40].

## Energy metabolism and oxidative stress

Energy generation and resultant oxidative stress are inexorably linked to inflammatory cell activation, especially via apoptotic signaling and NLRP3 inflammasome activation. Mitochondria produce approximately 90% of all endogenous reactive oxygen species (ROS) making them major regulators of cellular stress, synaptic plasticity, and calcium homeostasis [41]. Alterations in their structure or function, commonly seen in BD, can result in less efficient energy generation (more oxidative byproducts per molecule of adenosine triphosphate (ATP)) as well as impaired antioxidant capacity [42].

Tissue samples from bipolar patients reveal alterations in mitochondrial size, morphology, distribution, and membrane potential [41, 43, 44]. Likewise, patients with various mitochondrial diseases demonstrate up to a 20-fold higher incidence of BD phenotypes than the general population [45].

Mitochondrial respiratory complexes of patients with BD also tend to be decreased in concentration, activity, and mRNA expression. Specifically, mitochondrial complex I dysfunction results in decreased intracellular and extracellular BDNF levels, which could partially explain deficits in plasticity and neurocognitive decline seen in BD [46].

Compared to healthy controls, patients with BD commonly display decreased intracellular pH, elevated lactate levels (suggesting a shift towards anaerobic metabolism), decreased limbic N-acetyl-aspartate (NAA) (a surrogate marker for mitochondrial function and neuronal viability), and increased levels of nitric oxide levels (a highly reactive oxidant molecule), as well as evidence of increased oxidative damage to lipids, proteins, and nucleic acids [41, 42, 44, 47, 48].

Elevations of peripheral markers of oxidative stress also correlate with earlier age of onset, longer duration of illness, and increased frequency of manic/depressive episodes [26, 44, 49, 50]. Evidence also suggests that a reactive increase in antioxidant enzymes occurs early in the developmental course of BD, especially during depressive phases [51].This strongly implies that oxidative damage plays both an inciting and exacerbating role in BD, which is suggested to contribute to declines in cognitive functioning, progressive shortening of inter-episode intervals, reductions in treatment response, and increased cardiovascular mortality [26, 41, 44].

Lithium treatment has been associated with decreased markers of oxidative stress (TBARS, SOD, hydrogen peroxide, cingulate cortex lactate, lipid peroxidation), and increased mitochondrial complex I activity [49, 51–53]. Moreover, the mitochondrial DNA (mtDNA) 10398A mutation, which is associated with higher fasting glucose and lower glucose oxidation in the prefrontal cortex, also confers a better response to lithium treatment in patients with BD [41, 44]. This suggests that beyond its acute therapeutic mechanisms (discussed throughout this review) lithium provides a long-term, neuroprotective benefit to patients by reducing overall oxidative damage to neurons by ameliorating mitochondrial dysfunction [48].

In summary, it appears that mitochondria are altered in BD, even before clinical symptoms arise. Their impaired function leads to increased oxidative damage and subsequent immune cell and NLRP3 inflammasome activation [54]. Importantly, several NLRP3 inducers (ATP, uric acid (UA), and Ca^2+^) are also associated with loss of mitochondrial membrane potential, and treatment with mitochondrial complex I inhibitors decreases NLRP3-dependent IL-1β secretion [54]. Some evidence suggests that oxidized mtDNA (released during apoptotic programs) serves as a vital preamble to NLRP3 activation (discussed in Sect. 4), further linking these two systems [55].

## Calcium homeostasis and inflammation

Beyond mitochondrial/oxidative dysfunction, alterations in endoplasmic reticulum (ER)-related stress responses and calcium signaling appear to be some of the most consistent findings across clinical, cellular modeling, brain-imaging, post-mortem, and genome-wide association (GWAS) studies in BD [44, 56]. Like ROS, calcium appears to be a potent NLRP3 activator [54].

Ca^2+^ ions have been shown to modulate neuronal excitability, neurotransmitter synthesis/release, synaptogenesis, plasticity, and apoptosis. Interestingly, only a tiny fraction (< 1%) of total body calcium exists as free intracellular ions, but even small alterations therein can lead to large decrements in neuronal function and rapidly activate apoptotic cascades [41, 44, 56].

Several studies have identified common SNPs in voltage-gated calcium channel genes, most notably the subunit 1C (CACNA1C) locus in patients with BD [41, 44, 57]. Induced human neurons derived from high-risk BD genotype subjects show both increased CACNA1C gene expression and enhanced calcium signaling [58]. Importantly, this hyperexcitable phenotype is selectively reversed by lithium in vitro, but only in samples taken from patients that respond to lithium clinically [59].

**Fig. 2:**
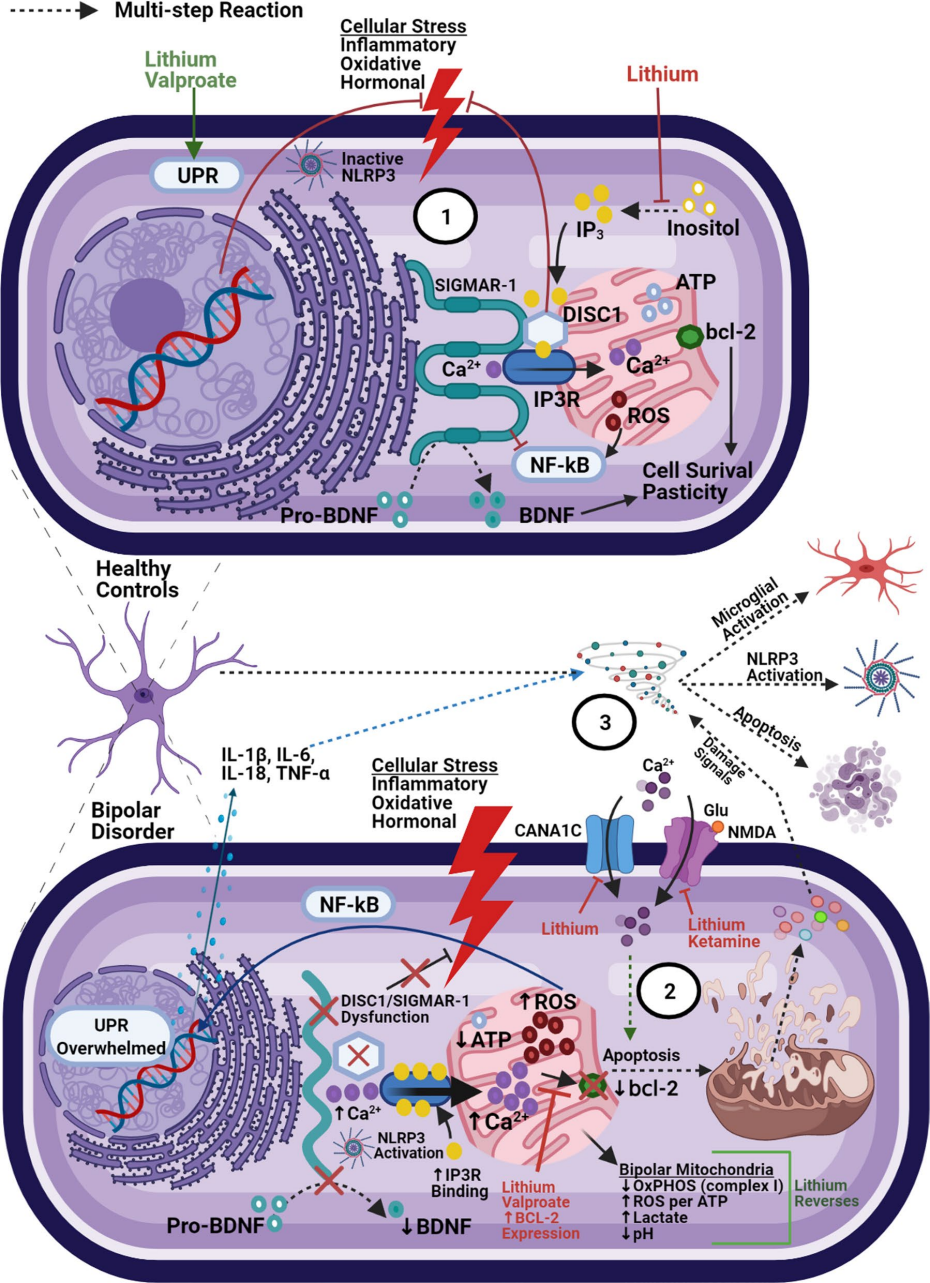
Oxidative damage and mitochondria-associated membranes. (1) Under normal conditions, ER stress activates the UPR which acts to restore homeostasis. Oxidative and hormonal stress stimulate ER-to-Mitochondria Ca^2+^ transfer (via inositol triphosphate receptors (IP3Rs)). MAM proteins (SIGMAR-1 and DISC1) act to stabilize IP3Rs and limit overall mitochondrial calcium accumulation. Independently, DISC1 acts to counteract corticosterone-induced stress (HPA-axis overactivity) and SIGMAR-1 acts to inhibit pro-inflammatory gene expression (NF-kB) and assist in the formation of mature BDNF. (2) Mitochondrial dysfunction in BD leads to increased oxidative damage, overwhelming the UPR. SIGMAR-1/DISC dysfunction can lead to decreased BDNF expression, loss of feedback on hormonal/oxidative stress signals, and increased IP3R ligand binding (subsequently increasing calcium influx into mitochondria). Risk SNPs associated with the CACNA1C locus can lead to even greater Ca^2+^ activity, further overwhelming anti-apoptotic signals (bcl-2). (3) Taken together, this dysfunction leads to increased pro-inflammatory signaling, NLRP3 inflammasome assembly at the ER-mitochondrial border, and eventually apoptosis, pyropoptosis, or autophagy. Subsequent release of cellular contents can cause amplification of extracellular inflammatory signaling and neurotoxicity (detailed in Figs. 3 and 4)

Mitochondria and endoplasmic reticula (ER) work together to maintain a delicate balance of intracellular [Ca^2+^] through lipid-raft-like subdomains called mitochondria-associated-membranes (MAMs), which link the two organelles. Notably, MAMs serve as a localization site for NLRP3 complex assembly [54]. Once assembled, MAM-NLRP3 complexes serve as detectors for increased ROS production from damaged mitochondria, subsequently leading to pro-inflammatory cytokine release [60] (Fig. 2).

**Fig. 3:**
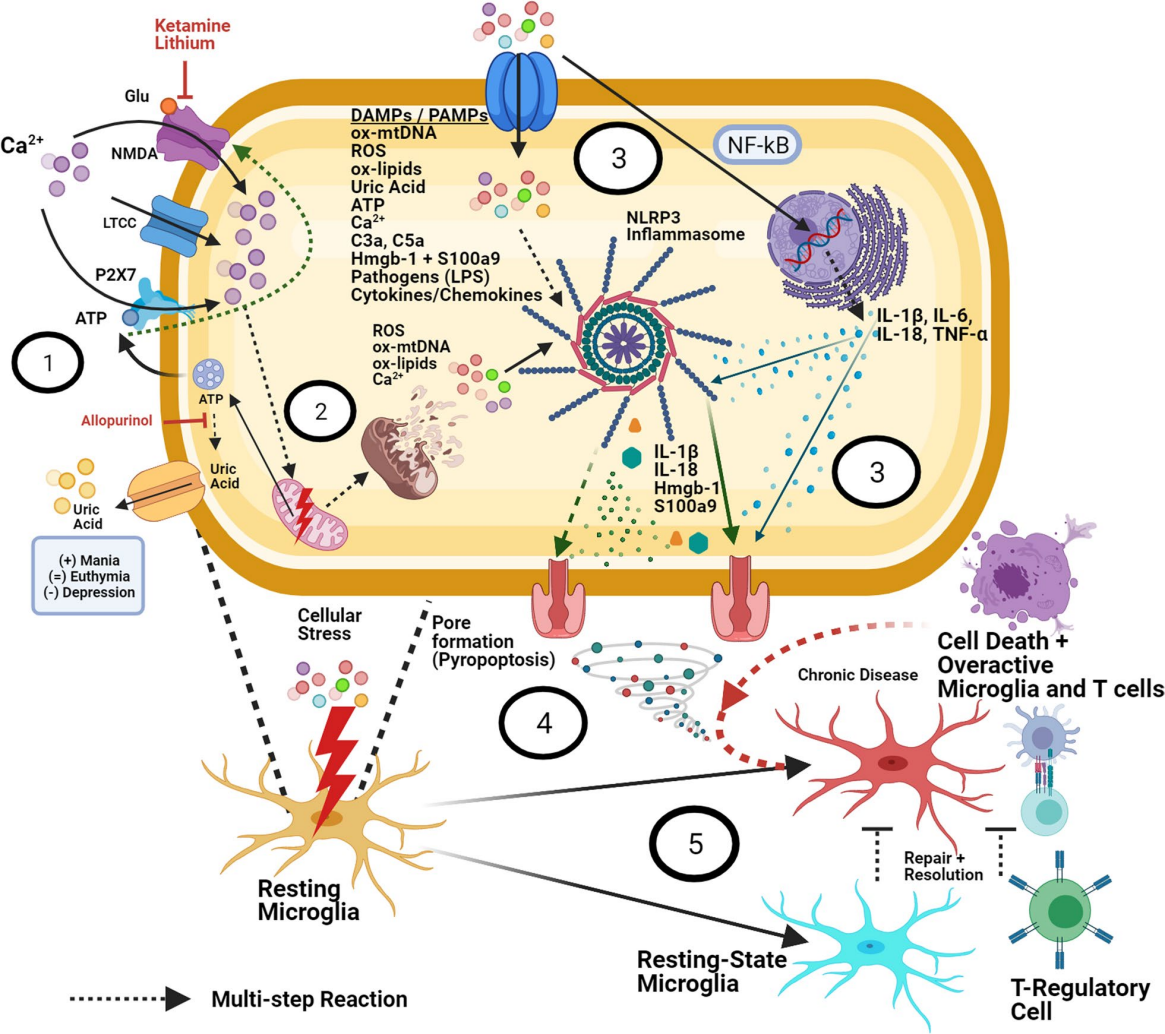
NL3P3 inflammasome activation. (1) Increased cellular stress causes the release of toxic metabolites (ATP, uric acid, glutamate, DAMPs, PAMPs, and others), increasing influx of extracellular calcium through P2X7, NMDA, and L-type calcium channels (LTCCs). ATP-P2X7 upregulation independently induces neuroinflammation (via NLRP3 activation, NF-kB, NFAT, GSK3β and VEGF signaling—not shown here), increases NMDA-excitotoxicity, and has been associated with rapid cycling in BD. (2) Mitochondrial dysfunction (decreased complex I activity) results in decreased oxidative phosphorylation and increased generation of ROS, Ca^2+^, as well as oxidized lipids and mtDNA. Intracellular Ca^2+^ imbalance can overwhelm deficient mitochondrial buffering capacity in BD, leading to fragmentation, morphological abnormalities, and eventually apoptosis. By any means, apoptotic signals (especially release of oxidized mtDNA), which potently upregulate NLRP3 activity [55]. (3) Damage signals activate the NLRP3 inflammasome, which (via caspase-1 cleavage) causes the elaboration of mature IL-1β and IL-18 as well as pore formation in the cell membrane (pyropoptosis), and ultimately the release of these deleterious intracellular molecules into the extracellular space. DAMPs/PAMPs also upregulate proinflammatory gene expression (NF-kB) and subsequent release of IL-1β, IL-6, IL-18, TNF-α, and other cytokines. (4) Once in the extracellular space, these agents act to amplify inflammation, activating surrounding microglia, increasing BBB permeability, recruiting peripheral immune cells, and upregulating the complement cascade (via Hmgb-1 and S100a9—MBL binding), causing sustained, sterile inflammation in the brain. Sublytic membrane attack complex (MAC) stimulation can also lead to increased mitochondrial calcium influx and loss of mitochondrial membrane potential, resulting in NLRP3 activation (not shown here) [54]. (5) In healthy individuals, these signals serve as repair mechanisms, and are deactivated by anti-inflammatory feedback (mainly from microglia and T regulatory cells), restoring homeostasis. In BD, chronic mitochondrial dysfunction and lack of proper feedback signaling results in amplification of inflammation and chronic neurodegeneration

Several MAMs have been associated with BD, including the disrupted-in-schizophrenia 1 (DISC1) protein [61] and the sigma-1 receptor (SIGMAR-1) [62]. DISC-1 plays a pivotal role in calcium signaling and mitochondrial trafficking, influencing several stages of neurodevelopment (synaptogenesis, neurogenesis, migration, and differentiation). DISC-1 also complexes with (and inhibits) GSK3β, mediating DNA repair from oxidative damage [63] and inflammatory cytokine expression [64]. Bipolar patients with the DISC1 risk haplotype demonstrate increased frequency and earlier onset of manic episodes compared to control subjects with the protective haplotype [61].

SIGMAR-1 appears to have a wide range of protein interactions, also mediating inflammatory signaling in microglia and astrocytes across various neuropsychiatric disease models [65]. It has been shown to counteract apoptotic/inflammatory signals (via inositol 1, 4, 5-trisphosphate receptor (IP3R), Bcl-2 and NFkB regulation) and enhance neuroplasticity (via BDNF expression) [41, 44, 62, 66–69]. It also acts as a key molecular chaperone in the unfolded protein response (UPR) system, which counteracts ER stress signals (ROS and hormonal). When the UPR is overwhelmed (as in BD), various elements including ROS, Ca^2+^, caspases, and others commit the cell to apoptosis or autophagy [56] (see Fig. 2). Unfolded protein accumulation also induces NLRP3 inflammasome activity [54].

Researchers have notably discovered a common SIGMAR-1 SNP associated with MDD and BD, especially in patients with mixed episodes, possibly suggesting a deeper mechanistic connection between both poles of the mood spectrum [62]. Initial findings suggest that the interplay between SIGMAR-1 signaling, oxidative damage, and inflammation could represent a significant link between affective disorders, CVD, and their mutual response to antidepressant therapy [70].

Overall, MAMs serve as a potent link between calcium signaling, oxidative stress, and inflammatory activation. They also appear as a common feature amongst psychotic, addictive, affective, and neurodegenerative conditions [69], possibly accounting for some of the phenotypic overlap across diagnoses. Several compounds with neuropsychiatric efficacy (lithium, valproic acid (VPA), haloperidol, fluvoxamine, fluoxetine, escitalopram, donepezil, ifenproil, and dehydroepiandosterone (DHEA)) appear to exert some of their positive effects via MAM/UPR pathways [67, 69–71]. Moreover, both lithium and VPA potently enhance antiapoptotic Bcl-2 expression [72]. Importantly, Bcl-2 expression significantly decreases IL-1β release and NLRP3 activation in response to several NLRP3 stimulators [54]. Both drugs also work to stabilize neurotransmission and increase hippocampal synapse formation by depleting inositol, a key calcium-signaling intermediary [73]. Further investigation to elucidate specific interactions between DISC1, SIGMAR-1, and the NLRP3 inflammasome may hold significant potential both therapeutically and diagnostically.

## Inflammasome activation

As described above, periods of excessive ER/mitochondrial stress can result in significant protein/calcium dyshomeostasis, triggering numerous downstream effects, including apoptotic and neuroinflammatory cascades. The NLRP3 inflammasome is a cytosolic protein complex composed of NLRP3, apoptosis-associated speck-like protein containing a C-terminal caspase recruitment domain (ASC), and the effector molecule caspase-1 [74]. In its inactive form, NLRP3 resides on the ER or in the cytosol, but appears to colocalize with MAMs upon stimulation with inducers [54, 60]. In a reciprocal manner, NLRP3 activity contributes to mitochondrial destabilization, subsequently releasing further NLRP3 activators [54].

NLRP3 inflammasome activation remains enigmatic. However, in many cases it appears to be a two-step process, first requiring priming via toll-like receptor binding and NF-kB-mediated pro-IL-1β expression [60]. A second activation step can occur through multiple effectors including the UPR, ROS, oxidized mtDNA, Ca^2+^, lipids, purines (especially uric acid and ATP), complement anaphylatoxins, β-amyloid, and pathogens. Collectively, these signals are referred to as pathogen-associated molecular patterns (PAMPs) or danger-associated molecular patterns (DAMPs) [74].

Recognition of these molecular signals activates caspase-1, which in turn, leads to extensive membrane pore formation (pyropoptosis) and promotes the maturation and release of proinflammatory cytokines (IL-1*β* and IL-18) [74]. Notably, macrophages deficient in mtDNA will still undergo apoptosis, but display severely-attenuated mature IL-1β expression, suggesting that the interaction of oxidized mtDNA with NLRP3 may have special relevance amongst the inflammasome-activating signals [55].

Regardless of activation route, pyropoptotic cell death releases a second series of DAMPs that can cause paracrine augmentation of the inflammasome response. Likewise, the elaboration of mature IL-1*β* and IL-18 increases the recruitment of additional inflammatory/effector cell populations. Importantly, depending on the nature of the insult, quantity of anti-inflammatory feedback, and functional status of endogenous restoration mechanisms, perpetuation of this inflammatory cycle can either culminate in damage repair or in progression of chronic disease (Fig. 3) [75].

Recent post-mortem analysis of frontal cortex samples from BD patients supports this energy-inflammatory hypothesis, revealing lower levels of mitochondrial complex I along with higher levels of mitochondrial NLRP3 and ASC and increased levels of caspase 1, IL-1β, IL-6, TNFα and IL-10 [76]. Moreover, NLRP3 and IL-1β expression are both regulated by multiple miRNAs that have been implicated in BD [77, 78]. CSF IL-1β concentrations are also elevated in patients who have had at least one hypomanic/manic episode in the previous year [79].

Notably, peripheral IL-1β levels in bipolar patients correlate positively with both suicide risk and leptin (a marker of insulin resistance) [80, 81]. This evidence strongly implicates the NLRP3 inflammasome as a nexus between oxidative damage, immune function, and cardiometabolic disease. NLRP3 activity has been extensively implicated in the pathophysiology of several metabolic disorders including obesity, type 2 diabetes, gout, and CVD, all of which occur more frequently in BD [82]. Furthermore, several NLRP3-interactive medications used in those conditions (statins, allopurinol, pioglitazone) have also displayed some efficacy in treating certain BD subpopulations/symptoms (statins for cognition in elderly BD, allopurinol for acute mania, and pioglitazone for bipolar depression) [82–85]. Growing genetic and preclinical evidence has further implicated inflammasome overactivation in many other BD-associated diseases including asthma, inflammatory bowel disease, multiple sclerosis, Alzheimer’s disease (AD), and Parkinson’s disease (PD), warranting further investigation [82].

In summary, the NLRP3 inflammasome serves a central mediator of inflammatory signaling in BD, linking other cellular stress systems to immune cell activation. It also provides a common thread between mood symptoms and metabolic comorbidities. While NLRP3 inhibition has been demonstrated with many common antidepressants [86], VPA [87], and clozapine [88], a significant portion of medications used in BD ultimately exacerbate metabolic syndrome. Determining the NLRP3 activity of mainstay treatments with poor metabolic profiles may be of significant relevance.

Future therapies focused on primary NLRP3 inhibition may also help to reverse this trend. For example, Baicalin (the active ingredient in the *Radix Scutellariae* plant) has shown strong biological activity and BBB penetration, being widely used to treat various infectious/inflammatory disorders, in certain countries [89]. Preclinical studies have shown that baicalin also exhibits antidepressant effects via GSK3β/NF-κB/NLRP3 signaling and HPA axis normalization, also mitigating obesity and insulin resistance in diabetic patients [89].

Ketogenic diets have repeatedly demonstrated efficacy in several forms of epilepsy, with many trials reporting significant improvements in mood, anxiety, sleep, cognition, and quality of life [90]. A recent randomized controlled trial further demonstrated that many of these improvements can occur independent of seizure control [91]. Proposed therapeutic mechanisms are diverse, including regulation of neurotransmitter signaling, mitochondrial function, insulin sensitivity, and inflammation [90]. Initial, evidence suggests that beta-hydroxybutyrate (BHB) (a primary ketone upregulated by the diet) can attenuate stress-induced behavioral and inflammatory responses via NLRP3 inhibition [92]. Positive impacts on mood, cognition, and weight loss have also been reported across several case studies in individuals with BD and schizophrenia [93]. Researchers at Stanford University have recently initiated the first open label trial to more rigorously investigate these findings in both disorders [94]. Though more quality research is needed, the majority of available evidence suggests a favorable safety and efficacy profile for patients with obesity and diabetes [95]. Such interventions may hold promise as adjunctive therapy in BD, especially in patients with high metabolic burden.

## Purines

ATP plays a central role in cellular homeostasis, linking most energetic and biosynthetic processes, and is one of the most conserved damage signals across mammalian species. It mediates both sterile and septic inflammation, as well as cell survival and proliferation across a wide range of chronic diseases and infections [96]. ATP and other purinergic metabolites (such as uric acid and adenosine) act as potent ligands for purinergic receptors on a wide array of immune cells, influencing chemotaxis, platelet granule release, and cytokine activity [96].

Overall, purinergic signaling has been shown to interact with most other neurotransmitter and second messenger systems involved in mood disorders, exerting control over synapse formation, plasticity, and virtually all stages of the glial-immune life cycle [96–99]. Indeed, purine dysfunction has been linked to a wide array of mood and anxiety symptoms including disturbed sleep, anhedonia, cognitive impairment, psychomotor agitation, severity of emotional symptoms, changes in appetite, and energy levels [98].

As the final product of purine metabolism, uric acid (UA) is itself an antioxidant compound, but also potently increases inflammatory cell activity, specifically via NLRP3 inflammasome-mediated IL-1β production. This mechanism has been observed in BD as well as associated conditions such as cardiovascular disease (CVD), gout, and other inflammatory illnesses [100].

Elevated UA—indicative of increased genetic turnover and/or cellular damage—has been consistently associated with acute manic episodes, and correlates with both symptomatic severity and improvement, as well as the development of CVD in BD [83, 100–102]. Even in the absence of a psychiatric diagnosis, elevated UA levels have been associated with specific manic traits such as impulsivity, irritability, increased drive, disinhibition, and hyperthymia [97, 103, 104].

UA abnormalities appear to be absent in euthymia [83] and subnormal levels have been associated with MDD and depressive mood scores in BD, independent of current disease phase [99]. Moreover, higher serum UA is predictive of bipolar conversion in currently depressed patients [105]. Remission from mania/hypomania is also associated with reductions in serum UA and enhanced urinary UA excretion [106]. Given the correlation between plasma and CSF UA levels, this metabolite may hold potential as a future state-dependent biomarker and outcome indicator in BD [107].

Conversely, adenosine has well-established anti-inflammatory effects across most immune cell populations [108]. Compared to healthy controls, bipolar patients also display lower serum adenosine levels. Adenosine orchestrates numerous excitatory (glutamate) neuronal/glial interactions in the CNS. Adenosine receptor agonists characteristically display anti-aggressive, anticonvulsant, and antipsychotic properties, whereas antagonists (caffeine, theophylline) can enhance manic symptoms such as irritability, anxiety, insomnia, and even psychosis [99, 109].

Adenosine signaling dysfunction has been associated with several autoimmune and inflammation-mediated conditions more prevalent in BD (diabetes, CVD, systemic lupus erythematous, multiple sclerosis (MS), rheumatoid arthritis, and others) [108, 110, 111]. Notably, a mainstay treatment for many inflammatory conditions (methotrexate), has been shown to exert its primary therapeutic effect via adenosine signaling [108].

Reinforcing this dynamic, allopurinol (a xanthine oxidase inhibitor that both increases adenosine and lowers UA formation) has repeatedly demonstrated efficacy as an anti-manic adjunct in BD and in treating impulsivity/aggression in elderly patients with dementia [112, 113].

The purine P2X7 surface receptor (an ATP-gated ion channel) has been unanimously associated with inflammation and is expressed on the majority of immune and CNS cell types (with the debatable exception of neurons and astrocytes) [1, 96]. Increased P2X7 expression has been demonstrated in AD, PD, MS, MDD, BD, and brain tumors [1]. Specifically, altered P2X7 expression has been implicated in the development of sleep deprivation and rapid cycling in BD [99]. In amphetamine-induced mania animal models, pharmacologic antagonism or genetic deletion of P2X7 appears to be behaviorally therapeutic, with concomitant reductions in proinflammatory cytokine release and lipid peroxidation [1].

In healthy individuals, basal concentrations of extracellular ATP are low (nanomolar range), and P2X7 acts a silent receptor, only activating under pathological states (such as the introduction of intracellular pathogens or release of ATP by dying cells) [1]. In diseases such as BD, various signals leading to chronic P2X7 overstimulation can cause deleterious inflammation and extensive tissue damage [96]. Downstream signaling from this receptor facilitates damage responses via several prominent pathways relevant to BD including NLRP3 inflammasome stimulation, proinflammatory cytokine release (TNFα, IL1-β, IL-18, IL-6, COX-2, CCL2, CCL3, and CXCL2), *N* ‐methyl‐d ‐aspartate (NMDA)‐induced excitotoxicity, and increased expression of NF-kB, NFAT, GSK3β, AKT and VEGF [99, 114]. (See Fig. 3 and the following reviews for more detail [1, 96]).

Though the precise mechanisms are incompletely understood, significant evidence suggests that chronic stress increases glutamate signaling in astrocytes, which causes the release of extracellular ATP and subsequent P2X7 receptor activation on surrounding microglia. ATP-P2X7 binding appears to be integral to NLRP3 inflammasome-driven IL1-β release, independently causing oligomerization of NLRP3 with ASC and pro-caspase-1 and subsequent induction of TNFα [115]. The ATP-P2X7-NLRP3 pathway seems to be a predominate mediator of stress-induced inflammation, holding great relevance for a wide range of neuropsychiatric (and systemic) illnesses [1].

P2X7 antagonism has demonstrated both antidepressant and anti-manic effects in animal models [116], with concomitant reductions in proinflammatory cytokine release (TNFα and IL1-β) [115]. In vivo antidepressant effects of several medications (including imipramine and ketamine) have also been associated with decreased hippocampal P2X7 receptor activity [1]. Early trials with P2X7 antagonists have been underwhelming across several conditions (cardiac allograft, hip fractures, osteoporosis, inflammatory bowel diseases), hampered by low selectivity and poor CNS penetration [1, 96]. Currently, several P2X7-selective antibodies are in early stages of development for MS, neuropathic pain, and AD [1, 96].

Recently, Johnson & Johnson has initiated the first phase II trial of a CNS-penetrant, selective P2X7 inhibitor in depression, notably recruiting only patients with baseline elevations in CRP [117]. Importantly, P2X7 sits upstream from the majority of inflammatory signals mentioned throughout this review, theoretically providing a more advantageous target compared to conventional anti-inflammatory treatments. Given its therapeutic potential in many common BD-inflammatory comorbidities, we suggest that P2X7 selective molecules warrant further investigation for bipolar disorder.

Overall, it appears that purinergic signaling powerfully mediates mood symptoms in BD, with some metabolites increasing (uric acid, ATP) or reducing (adenosine) inflammation. Via modulation of immune function, this system presents a strong mechanistic link between BD and many of its inflammatory comorbidities. As such, aspects of this system (particularly P2X7) hold promise as not only biomarkers, but therapeutic ingresses necessitating more selective/penetrant development going forward.

## Kynurenine pathway

Tryptophan (TRP) metabolism through the kynurenine (KYN) pathway represents yet another significant interface between cellular stress and systemic inflammation in BD, having wide-ranging effects. Neuroactive byproducts of this pathway modulate immune, neurotransmitter, endocrine, metabolic, and hormonal activity [118, 119]. Under normal conditions, 95% of free TRP is converted to KYN via two enzymatic pathways: intra-hepatic tryptophan dioxygenase (*TDO*) or extra-hepatic (CNS, lungs, small intestine, immune cells) indoleamine 2,3-dioxygenase (*IDO*).

**Fig. 4:**
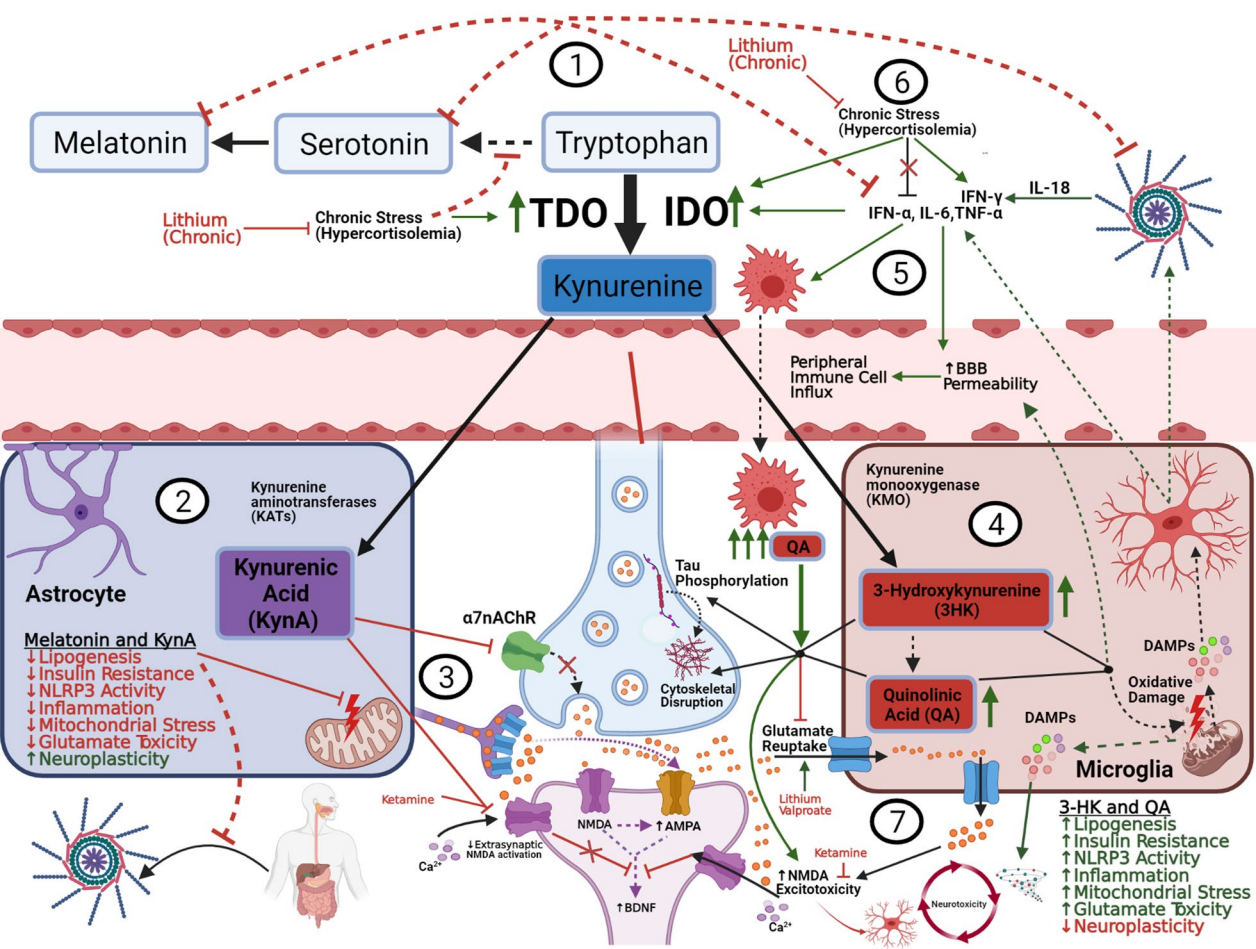
The Kynurenic pathway, glutamate, and neuroplasticity. (1) 95% of free, extracellular tryptophan is converted into KYN via IDO and TDO. BD, stress, and metabolic disease augment this conversion, decreasing free serotonin/melatonin with resultant increases in oxidative/inflammatory signaling. (2) Inside astrocytes, KYN is converted into KynA, which exerts a net neuroprotective effect by several mechanisms. (3) Through extra-synaptic NMDA-inhibition, ketamine shunts glutamate towards AMPA receptors and blocks QA influx, ultimately upregulating BDNF-mediated synaptic plasticity, in concert with an increase in KynA/QA ratio (NMDA receptor-kynurenic competition). (4) Inside microglia, KYN is converted to 3-HK and QA which exert various neurotoxic effects. (5) Proinflammatory cytokines increase QA production by increasing IDO activity and shunting tryptophan into microglia. This in turn amplifies the inflammatory signal, causing influx of peripheral immune cells, which exponentiate QA production and subsequent toxicity. (6) Under chronic stress (HPA overactivity), cortisol loses its feedback inhibition on pro-inflammatory signals, and continues to upregulate QA production though TDO/IDO induction and IFN-γ modulation, exacerbating the neurotoxic/inflammatory environment (discussed in Sect. 7). (7) Astrocytic processes form a “cradle” (left side) around synaptic connections and serve as the major site for glutamate reuptake, limiting excess glutamate spillover into extrasynaptic NMDA receptors. Astrocytes also exert negative feedback (not shown) to suppress microglial overactivation. Chronic inflammation, exacerbated by acute mood episodes (right side) disrupts the astrocytic cradle and impairs feedback mechanisms, resulting in extracellular glutamate accumulation and microglial overactivation. Activated microglia do engage in modest glutamate reuptake, but it is suppressed by QA and outpaced by their immune-stimulated release of various toxic metabolites (QA, ROS, Glutamate, cytokines, damaged organelles, etc.). Extrasynaptic NMDA activation suppresses BDNF expression, impairing synaptic homeostasis. Ultimately these signals result in a chronic reciprocal exacerbation of glutamate excitotoxicity, inflammation, and overall neurotoxicity (discussed in Sect. 8)

While both enzymes are regulated by stress hormones (glucocorticoids), IDO function appears to be significantly more responsive to both proinflammatory mediators and psychosocial stress [119]. As follows, euthymic bipolar patients display increased TRP to KYN conversion overall, compared to healthy controls [120]. This conversion becomes more pronounced once inside the CNS, where KYN exerts differential effects depending on whether it is metabolized by astrocytes (neuroprotective) or microglia (neurotoxic). Notably, inflammation and stress not only augment TRP to KYN conversion (via IDO upregulation) but also increase levels of neurotoxic KYN metabolites (due to enhanced microglial over astrocytic throughput) [118]. (See Fig. 4).

Astrocytic metabolism converts KYN into kynurenic acid (KynA). At physiologic levels, KynA acts as an ROS scavenger, inhibits dopaminergic neurotransmission, and antagonizes NMDA and nicotonic (α7nAChR) receptors [118, 119, 121]. Blockade of these receptors results in both decreased presynaptic glutamate release and increased reuptake of excess glutamate by microglia and astrocytes. This is suggested to reduce broader excitotoxicity and inflammatory signaling, thus modulating synaptic plasticity and cognition [119]. Peripherally, KynA has been shown to reduce stress-induced colitis in mice by promoting NLRP3 inflammasome autophagy, demonstrating the pervasive impact of this metabolite [122]. (See Wirthgen et al. for a detailed description of the immunomodulatory properties of KynA) [121].

Conversely, microglial KYN processing results in the formation of quinolinic acid (QA) and 3-hydroxykynurenine (3HK), which have been linked with various neurotoxic processes (NMDA agonism, glutamate reuptake inhibition, ROS generation, cytoskeletal destabilization, BBB disruption, tau phosphorylation, and proinflammatory mediator upregulation) [123].

Proinflammatory conditions can severely exacerbate kynurenic toxicity by increasing BBB permeability leading to influx of peripheral immune components that amplify and sustain cytokine release. Numerous studies indicate that proinflammatory cytokines (IFN-α, IL-6, TNF-α, and especially IFN-γ) induce IDO production from immune cells, thus increasing microglial QA production [119]. Importantly, IL-18 (released from NLRP3) is a potent inducer of IFN-γ, thus linking inflammasome activation to kynurenine dyshomeostasis [26].

Moreover, with increased BBB permeability, peripheral macrophages enter the CNS, producing roughly 30 times more QA than typical CNS microglia and release copious amounts of glutamate into the extracellular space, causing extensive, global damage. Post-mortem samples from bipolar patients support this mechanism, showing increased IL-6, TNF-α, and QA in the frontal cortex [119]. Interestingly, initial evidence suggests that kynurenine toxicity may be driven by different primary cytokines in mania (TNF-α) versus chronic bipolar depression (IFN-γ) [124], though further confirmation is needed.

Possibly owing to the high density of NMDA receptors, lower KynA/QA ratios have been repeatedly associated with reduced hippocampal volume and functionality in MDD, BD, and concussed athletes with depressive symptoms [118]. Compared to healthy controls, serum KynA levels are significantly lower in MDD, psychotic BD, and schizoaffective disorder, with the later showing the largest decrement [118]. Suicide attempts are also associated with a neurotoxic shift of the KynA/QA ratio in both CSF and plasma for up to two years after the attempt [118].

Amongst bipolar patients, increased TRP to KYN conversion appears to be more pronounced with comorbid obesity, suggesting metabolic inflammation may exacerbate BD symptoms via kynurenine signaling [120]. Preclinical studies support this notion. IDO-knockout mice and those with elevated KynA are protected against the obesogenic, inflammatory, and insulin resistance-inducing effects of high-fat diets. Conversely, T2DM has been associated with reduced Kyn/QA ratios, and prolonged QA elevation increases the risk of developing T2DM [118].

LPS-induced depression in mice can be blocked by both genetic and pharmacological inhibition of IDO. Interestingly, Walker et al. demonstrated that, while LPS increases CNS QA, depressive effects can also be blocked by low dose ketamine, without altering immune/inflammatory function or IDO activity. This suggests that KynA/QA competition at the NMDA receptor may represent a final common pathway for the glutamate and inflammatory models of depression [125]. Through NMDA antagonism, ketamine is also theorized to work by shifting glutamate into the AMPA-receptor pathway, which upregulates BDNF-mediated synaptic protein synthesis (see Sect. 8 for more detail) [119]. As such, ketamine has demonstrated rapid antidepressant efficacy in refractory BD, with a concomitant neuroprotective shift in kynurenine metabolites [119].

Recent work has drawn attention to another kynurenic-inflammatory mediator, the aryl hydrocarbon receptor (AhR). KYN is one of many endogenous ligands for this receptor, whose activation portends central and peripheral inflammatory responses across most immune cell lines (especially via NLRP3 and GSK-3β stimulation) [26]. Once ligand-bound, the AhR undergoes nuclear translocation with wide ranging effects on many target genes. Notably, AhR complexes alter cytochrome P450 expression, thereby altering mitochondrial function as well as medication and hormone metabolism (discussed in more detail in the next section). Preliminary evidence also suggests this receptor may profoundly regulate GI-immunological homeostasis, mucosal integrity, and adipogenesis [26].

Toxin-induced activation of AhR may also cause disruptions in calcium and zinc homeostasis, both of which have been implicated in BD. Of note, cigarette smoke, which has been independently associated with disease severity and suicidality in BD, contains many AhR activating toxins [126].

Several current therapies (ECT, physical exercise, COX-inhibitors) have demonstrated efficacy in BD and MDD associated with neuroprotective shifts in kynurenine metabolite profiles [118]. Looking forward, two clinical trials in MDD are currently investigating a KynA analog and leucine, which is reported to block KYN transport across the BBB [127, 128]. Likewise, IDO-inhibitors are being developed to treat various malignancies (which have inflammatory components) [129]. Given their central function in the pathway, clinical inquiry in BD seems prudent, however current obstacles exist in clinical application [130]. Significant potential notwithstanding, clinicians must proceed cautiously, as KYN modulation may have unanticipated effects in patients with cognitive and psychotic symptoms [118, 131].

## Hormones and circadian rhythm

Cortisol, melatonin, serotonin, dopamine, and norepinephrine all have well established, vital roles in mood regulation [26, 132] and immune signaling [133]. Aberrant expression of these hormones has been consistently associated with most prominent features of BD, particularly manic switching, suicidality, and treatment response [134, 135]. Patterns of their metabolism and activity display significant variability in response to oxidative/psychosocial stress, circadian periodicity, and the overall inflammatory milieu.

Melatonin itself is released by many, if not all mitochondria-containing cells, and exhibits significant antioxidant, anti-apoptotic, anti-excitotoxic (NMDA antagonist), and anti-inflammatory effects both in vitro and in vivo [26, 136]. Melatonin inhibits the expression of many KYN/AhR-induced genes, especially in the cytochrome P450 family, effectively preventing its own metabolism, further reducing mitochondrial oxidative stress [26]. Importantly, melatonin is produced in a 100-fold greater quantities within the GI-tract and acts as a significant inhibitor of gut permeability, in part by reducing NLRP3 inflammasome activity [26].

Conversely, pro-inflammatory cytokines (especially TNF-α) independently act to depress pineal melatonin synthesis and desynchronize CNS circadian genes [132]. As follows, BD patients characteristically display a delayed onset and a lower evening peak of melatonin secretion, as well as hypersensitivity to light-induced melatonin suppression compared to healthy controls [132].

Though it has failed to display efficacy in treating mood symptoms, exogenous melatonin has been shown to improve sleep onset latency and total sleep time in BD [137]. A recent meta-analysis of five randomized trials (limited sample sizes) also suggests that exogenous melatonin may improve antipsychotic-induced metabolic symptoms in bipolar and schizophrenic patients [138].

In healthy individuals, cortisol displays the opposite circadian pattern to melatonin: peaking early in the morning and lowest at night. Circulating cortisol exerts a negative-feedback effect at multiple levels within the hypothalamic–pituitary–adrenal (HPA) axis, which has been hypothesized as one of the key mechanisms associated with immune dysfunction in BD [139]. However, this relationship appears to be bidirectional and highly complex.

Glucocorticoids as a class display both anti-inflammatory (decreased IL-6/TNF-α) and pro-inflammatory (increased NLRP3 and NF-kB expression) effects. Under normal conditions, pro-inflammatory cytokines (IL-1, IL-6, TNF-α) directly stimulate the HPA-axis, resulting in increased glucocorticoid release, which in-turn suppress further pro-inflammatory factor production [140]. However, excessive inflammation (as in BD) can disrupt normal hypothalamic glucocorticoid receptor feedback-inhibition on cortisol production, resulting in sustained HPA-axis hyperactivity and further inflammation [139, 140]. Cortisol itself is also a potent TDO inducer, and likely contributes to adrenergic upregulation of IDO via IFNγ signaling, thus enhancing kynurenic toxicity. This relationship is likely bidirectional, as animal studies suggest KYN effectors such as the AhR independently regulate cortisol expression [26].

This possibly explains why BD patients display elevated basal cortisol levels, which are present during all disease phases, including into remission [141–143]. Upstream, alterations in corticotropin-releasing hormone (CRH) secretion have been shown to precede the clinically observable onset of manic or hypomanic symptoms, possibly holding promise as a future state-dependent biomarker [144]. Paralleling inflammatory behavior in BD, this evidence suggests that aberrant stress responsiveness may be a chronic feature which becomes heightened during acute mood episodes. Importantly, offspring of bipolar patients exhibit basal hypercortisolemia compared to those with no parental history of mental illness [145]. The heritability of basal cortisol secretion level is estimated to be 60% [146]. Thus, as with abnormalities in mitochondrial and immune cell function, HPA axis alterations likely contribute to the high degree of heritability in BD. While acute effects are less clear, long term lithium therapy tends to result in reduced basal cortisol levels, which appear to correlated with reductions in depression scores [147].

## Glutamate signaling and neuroplasticity

Virtually all psychiatric disorders demonstrate aberrancies in synaptic formation, remodeling, and destruction–-collectively termed neuroplasticity. Substantial evidence implicates glutamate (especially via AMPA and NMDA receptor activity) as a key mediator of dendritic spinal trafficking that underlies this process [148]. From early neurogenesis to formative synaptic pruning, and eventually neurodegeneration, immune signaling is inexorably linked to every facet of neuroplasticity and glutamatergic metabolism [149].

Under pathologic conditions (ischemia, oxidative stress, trauma, etc.) ROS and proinflammatory mediators can impair clearance (reuptake) and stimulate release of CNS glutamate. Prolonged excess of extracellular glutamate results in overactivation of extrasynaptic NMDA receptors, which suppresses the synthesis and release of BDNF and other neuronal growth factors. This ultimately results in atrophy of dendritic spines/processes, and eventually neuronal loss (Fig. 4). Moreover, an imbalance in the ratio of synaptic and extrasynaptic glutamate concentrations can cause aberrant ionotropic receptor activation, leading to loss of fidelity and specificity of local neurotransmission. Researchers suggest that this “noisy” regional circuit dysfunction may contribute significantly to the outward behavioral symptomatology of affective disorders [149].

Individual cytokine activity extensively modulates glutamate metabolism and neuroplasticity. Several promoter regions for glutamate reuptake receptor genes are responsive to immune signaling molecules (such as TNF-α and NF-kB) [150]. NLRP3 inflammasome-mediated IL-1β release has also been shown markedly increase NMDA receptor expression and overactivation [149]. IFN-*γ* controls astrocytic transition between neuroprotective and neurotoxic phenotypes (discussed below) and induces the release of toxic glutamate-like compounds such as QA (discussed in Sect. 6). ATP-P2X7 binding has also been shown to independently increase glutamate release from microglia [149].

On a cellular level, communication between neurons, glial cells, and astrocytes act to regulate synaptic glutamate homeostasis and neuroplasticity, modulating reuptake and conversion into glutamine for vesicular redistribution to neurons. Astrocytes handle the majority (80%) of glutamate reuptake, further providing a functional barrier (“astrocytic cradle”) around synapses, limiting spillover of excess glutamate (Fig. 4). Moreover, microglia serve as the primary site for conversion of glutamate into glutathione, the brain’s primary antioxidant [151].

Indeed, CNS tissues are far more susceptible to oxidative damage and inflammation compared to other organs, owing to their high metabolic activity and low antioxidant capacity. Glutathione is a tripeptide of glutamate, cysteine, and glycine, which depends on functioning glutamate reuptake for precursors. Glutathione depletion is tightly linked to microglial overactivation. Increased oxidative stress and inflammation not only reduce astrocytic glutamate reuptake activity but enhance microglial glutamate release, resulting in a self-perpetuating cycle of excitotoxicity and reduced antioxidant activity (Fig. 4) [152].

Though caveats and complexities exist, astrocytes and microglia can generally adopt proinflammatory/damaging (A1/M1) or anti-inflammatory/restorative (A2/M2) phenotypes depending on local cytokine profiles [149]. It appears that only activated microglia express glutamate reuptake transporters, however in response to damage signals discussed throughout this review (including glutamate itself), microglia appear promote significant neurotoxicity by releasing ROS, proinflammatory cytokines, and glutamate. Such conditions can also lead to a phenotypic transition in astrocytes, adopting phagocytic/antigen-presenting activity with upregulation of major histocompatibility complex (MHC) class II and NLRP3 inflammasome expression. Beyond compounding the proinflammatory milieu, such conversion can result in the loss of the “astrocytic cradle”, further impairing glutamate reuptake and increasing spillover/excitotoxicity (Fig. 4) [149].

Under normal conditions, astrocytes provide a negative feedback mechanism, secreting IL-10 and TGF-β in response to microglial inflammatory activation. Notably, aged astrocytes display a reduced capacity to restrain microglial activation [153], suggesting an omnidirectional exacerbation between chronic inflammation, accelerated cellular aging, and impaired neuroplasticity, all of which are characteristic of neuroprogression in bipolar patients.

T cells also appear to play a significant role in this dynamic, though current knowledge is limited compared to other cell types. Across several neurodegenerative animal models, T cells appear to display anti-excitotoxic, neurogenerative, and pro-cognitive effects [154]. T cells express NMDA and glutamate receptors which exhibit a dose-dependent response to extracellular glutamate. Low to normal physiologic concentrations (pico-nanomolar) appear to decrease excitotoxicity via astroglial modulation and neuronal growth factor secretion [154]. Higher glutamate concentrations (millimolar) seen in a variety of neuropsychiatric conditions appear to decrease T cell proliferation and increase proinflammatory cytokine (IFN-γ) release [154]. The anti-inflammatory cytokine IL-4, which is known to increase astrocytic BDNF expression, has been implicated in this dynamic. IL-4 knockout mice display significant deficits in learning/memory tasks, which can be reversed by allogeneic, wild-type T cell infusions (along with a shift towards the protective M2 phenotype in CNS myeloid cells). However such improvements are not seen with infusions of autologous T cells deficient in IL-4 receptors [154].

Glutamate displays extensive mutualistic interactions with neuronal growth factors. For example, glutamate increases the transcription and release of BDNF, which likewise enhances presynaptic glutamate release and independently potentiates NMDA receptor activation [155]. Following repeated excitatory (glutamate) transmission, BDNF expression ultimately leads to the development or strengthening of individual neuronal connections (long term potentiation) via several pathways discussed below. Moreover, BDNF modulates GSK3β/Wnt and PKC activity, both of which have been extensively implicated in BD pathogenesis (see this review for more detail) [156]. Though the interplay between glutamate and inflammation has been extensively investigated, immunomodulatory effects on BDNF signaling are less clear. In mice, both excessive immune suppression and activation appear to impair hippocampal BDNF expression, and local BDNF administration appears to enhance inflammatory myocardial damage [157–159].

With regards bipolar disorder, reduced peripheral BDNF levels (and mRNA expression) have been demonstrated during depressive and manic episodes [160, 161], also associated with cognitive dysfunction [161, 162]. BDNF levels appear to normalize during euthymic periods [161]. Genetic and epigenic association studies have produced inconsistent results in BD, especially with regards to the Val66Met BDNF polymorphism which generated initial transdiagnostic interest, but has since failed to garner consistent associations across subpopulations in BD [161].

Conversely, microRNA (miRNA) expression is emerging as a more promising area for genomic research. Neuronal miRNAs account for 70% of all miRNAs in the human body and are primarily involved in regulating neurogenesis and neuroplasticity. MiR-132 is the most replicated microRNA across all major psychiatric disorders. It is primarily involved in modulating BDNF-mediated neuronal differentiation but has also been implicated in glutamate receptor expression and anti-inflammatory signaling [163, 164]. See Gruzdev et al. for a complete review [163].

In that regard, major psychiatric disorders (BD, MDD, and schizophrenia) share significant phenotypic commonality, all potentially espousing depressive, anxious, psychotic, cognitive, psychomotor, and/or catatonic features—traits which have all been associated with glutamatergic dysfunction [149, 152, 165, 166]. Though current therapeutic mainstays primarily focus on monoaminergic modulation, glutamatergic neurons make up 60–80% of total brain metabolic activity, underscoring the need for further therapeutic development in this system [167]. Patients with all three disorders show differing, region-specific alterations in glutamate activity which have been associated with concomitant alterations in regional neuro-dendritic density/complexity. As a simplification, chronic MDD and schizophrenia appear to display reductions in cortical glutamate activity, while studies in BD have demonstrated mixed results, likely due to differences in mood phase and wider array of pharmacotherapies used in this population [165]. Caveats to these observations exist which are beyond the scope of this article, see the following reviews for more detail [163, 166, 168].

Notably, NMDA receptor potentiation produces efficacious therapeutic effects in schizophrenia, importantly targeting negative symptoms and cognitive dysfunction which tend to be refractory to conventional antipsychotics [148, 166]. In affective disorders, several antidepressant classes (TCA, SSRI, SNRI) also work in part by reducing NMDA and AMPA receptor subunit expression [148]. It is important to note that both hypoactive and excessive glutamate signaling can lead to dendritic spine loss, likely accounting for some of the heterogeneity of glutamate function across disorders and therapies [148]. Lastly, all three disorders share several candidate genes, including DISC1, which regulates dopamine signaling, neurodevelopment, dendritic spine density, mitochondrial trafficking, and glial cell activity, providing an important mechanistic link between inflammation, oxidative stress, and neuroplasticity across diagnoses [169].

Ketamine provides several other transdiagnostic links between inflammation and neuroplasticity. It was originally developed as a safer structural analog of phencyclidine (PCP) with less potent NMDA antagonist activity for anesthesia [170]. When administered in high doses, both compounds reliably mimic many of the cognitive and positive symptoms of schizophrenia [148]. Briefly, acute psychosis in schizophrenia is widely believed to result from NMDA receptor hypofunction on inhibitory GABA interneurons, leading to disinhibition (increased glutamate signaling) in layer V cortical pyramidal neurons [152]. This same pathway is suggested to comprise part of ketamine’s acute antidepressant mechanism of action (discussed below).Unlike the acute effects of ketamine however, prolonged overactivation of this pathway in schizophrenia is suggested cause chronic excitotoxicity, eventually resulting in glutamate hypofunction and neurodegeneration. Likewise, chronic administration of ketamine or PCP mimics many of the cognitive and negative symptoms of schizophrenia in animals [167]. These findings strongly implicate glutamate dysfunction in both mood and psychotic symptomatology.

With regards to affective disorders, ketamine has demonstrated robust efficacy in rapidly treating both bipolar and unipolar depression, especially in patients with suicidal ideation and treatment resistance [168]. At subanesthetic doses, its primary antidepressant efficacy has been linked to induction of a prefrontal “glutamate surge” which (in concert with antagonism of NMDA receptors) shunts glutamate through neighboring AMPA receptors (the so-called “NMDA to AMPA throughput” model; see Fig. 4). AMPA receptor stimulation subsequently increases expression of BDNF, which binds to intracellular TrkB receptors, ultimately upregulating the mechanistic target of rapamycin (mTOR) and other growth factors to induce or maintain synaptogenesis [171].

Rapamycin itself is an mTOR inhibitor, which inherently confers potent immunosuppressive properties. As one may assume, it has repeatedly been shown to prevent the anti-depressant and neuroplastic effects of ketamine in animal models [171]. In humans however, a recent first-in-class study by Abdallah et al. found that rapamycin pre-administration actually prolonged the antidepressant effects of ketamine [171].

Though these findings require substantial further validation, they deserve significant attention for the purposes of this review. First, mTOR signaling plays an essential role in cellular/neuronal autophagy, which has been implicated as a therapeutic mechanism of many antidepressants [172]. By removing toxic/damaged cellular components, mTOR-mediated autophagy prevents excessive NLRP3 inflammasome activation, limiting the spread of damage signals to surrounding areas. Second, rapamycin’s potent anti-cytokine properties may be synergistic with the acute effects of ketamine (some of which are plasticity-independent) [173]. In other words, the acute benefits of ketamine may be sustained via reducing pathologic inflammatory subversion.

Ketamine itself displays complex immunomodulatory characteristics, best described via its analgesic capacity, where doses are comparable to those used in depression. It appears to be most efficacious in pain types with a tonic-inflammatory component (neuropathic, cancer, etc.) [174]. Much like its antidepressant effects however, its analgesic benefits appear short lived, and utility in chronic pain management remains controversial [174].

This has, in part, led experts to describe ketamine as an “homeostatic immune regulator”, rather than a purely anti-inflammatory drug [175]. In other words, it appears to prevent exacerbation/extension of local inflammation without causing broader immunosuppression that can interfere with the “completion” of an inflammatory cascade. Unlike many neuropsychiatric disorders (including BD), where broad reduction of excessive/chronic neuroinflammation appears to be therapeutic, full elaboration of systemic, pro-inflammatory signaling is often necessary to promote successful tissue repair and treat the source of a patient’s pain [175]. It is suggested that this unique immunomodulatory profile contributes significantly to ketamine’s high clinical utility in acute pain management, perioperative, and critical care settings [174, 175].

In those environments, this phenomenon of incomplete immune cycle signaling is termed “immune-paralysis”, and can have deleterious long-term consequences [175]. It is believed to be largely mediated by an imbalance in Th1 (proinflammatory) and Th2 (anti-inflammatory) cell populations [175]. Gao et al. demonstrated that while ketamine and morphine both reduce overall T-helper cell differentiation in response to inflammatory stimuli, morphine potently decreases the Th1/Th2 ratio, whereas ketamine increases the relative concentration of Th1 cells [176]. Such findings are in accordance with broader themes of in vitro ketamine research. Namely, ketamine seems to have little impact on cytokine expression unless in the presence of an inflammatory stimulus. In such cases, it appears to modulate exclusively pro-inflammatory cytokine activity [175]. This is particularly relevant in the context of BD, given that so many of the processes described in this review entail a chronic loss of negative feedback by anti-inflammatory signals (see Figs. 3 and 4).

As follows, ketamine’s anti-inflammatory function in affective disorder patients remains elusive. Temporal associations between inflammatory changes and treatment response are at best, unclear [177–182]. Likewise, elevation in baseline inflammation appears to predict treatment response in some studies [177, 180] but not in others [178, 179, 183]. Given the timescale required to observe tangible effects with immunomodulation in general, it is probably not surprising that many studies fail to correlate rapid antidepressant onset with immediate reductions in cytokine activity. Indeed, ketamine (and its metabolites) espouse numerous non-inflammatory mechanisms which can account for their immediate antidepressant effects [184].

Thus, rather than focusing on the role of inflammation in initial treatment response, it would seem prudent to delineate the contribution of immune signaling to ketamine’s *inability to sustain* anti-depressant responses. Classically, elevated baseline inflammation is associated with treatment resistance to most antidepressant strategies, apart from ECT and targeted anti-inflammatory medications, where the inverse is true [32, 182]. Unlike those therapies, ketamine appears to temporarily modulate (rather than purely suppress) inflammation. Taken in the context of recent findings by Abdallah et al. it is conceivable that ketamine induces a wide array of positive adaptations, which are subsequently degraded when patients regress into their basal pro-inflammatory state.

Emerging evidence with other rapid-acting antidepressants may provide further support for this hypothesis. Specifically, serotonergic hallucinogens (SH) appear to provide potent antidepressant responses starting the first day after treatment, even in refractory populations, though broader clinical validation is needed [185]. Currently, a phase I trial using psilocybin in bipolar II depression is underway at the University of Maryland in The United States [188].

Many mechanistic similarities exist between ketamine and SHs. Both trigger a “glutamate surge” in layer V pyramidal neurons (albeit via direct pyramidal serotonin-2A receptor stimulation as opposed to intraneuronal NMDA blockade) [187]. Layer V AMPA receptor throughput is also necessary to produce their behavioral effects and sustain glutamatergic activity across animal models [188]. Both compounds also appear to exert part of their antidepressant activity via serotonin signaling [189].Similar patterns of downstream neuroplastic growth factor upregulation (particularly BDNF) have been reported, though SHs appear far more potent in that regard. With a few exceptions, in human studies, increasing levels of BDNF appear to correlate with acute antidepressant effects for both ketamine and SHs as well [185].

Unlike the short-lived effects of ketamine however, SHs have been reported to induce responses which last six months or more after just one to two sessions [185]. While superior growth factor stimulation may contribute to this phenomenon, repeated acute-phase administration of ketamine still confers relapse in the vast majority of patients within the first month following their final infusion [190]. Thus, we suggest that other mechanisms (including pathologic inflammatory regression) may contribute significantly to discrepancies in response duration.

In contrast to ketamine, SHs as a class exhibit potent anti-inflammatory effects across a myriad of disease models and cell lines [191–194]. The most studied SH with regards to inflammation, 2,5-dimethoxy-4-iodoamphetamine (DOI), has been shown to profoundly suppresses TNF-α mediated inflammation in rodent tissues at picomolar concentrations—equating to a dose in humans at least two orders of magnitude below what is necessary to produce hallucinogenic effects [185, 193, 194]. With the exception of a few natural toxins (i.e. botulinum), no compound has demonstrated comparable immunosuppressive potency [194]. Given that successful antidepressant responses with SHs necessitate doses sufficient to produce substantial hallucinatory effects, it is likely that their anti-inflammatory capacity in vivo may be profound. Though one recent trial has demonstrated CRP reductions which correlate with initial response [195], no clinical trials to date have provided longitudinal tracking of inflammatory markers after SH administration. We suggest that such investigation is highly warranted to assess whether sustained reductions in inflammation contribute to the characteristic antidepressant durability of this class. Likewise, determining whether ketamine’s effects are prolonged with repeated dosing of immunosuppressive agents may also yield substantial benefits for patients.

## Conclusion

BD exacts a high societal and individual burden. Current first-line treatments, while helpful for many patients, fall short of ideal results. Clearly, heterogeneity and lack of objective diagnostic markers significantly hamper progress. Individual variation in the expression of countless pathophysiologic mechanisms necessitates a re-assessment of conventional approaches.

Inflammatory signaling draws a common thread between so many of these disparate disease processes. Notably absent from this review are the effects of the microbiome, which appear to mediate a significant portion of the inflammatory signals discussed here. Though in its relative infancy, we anticipate that recent improvements in complexity analysis will further solidify the importance of inflammatory mechanisms in BD.

While excess inflammation is not ubiquitous in BD, it does present an opportunity to objectively segregate a large subset of patients who may respond better to targeted therapies. Moreover, addressing excess inflammation early in the disease course may yield substantial improvements in ameliorating neuroprogressive features and comorbid conditions. Ultimately, enhanced targeting of inflammation may produce improvements in functional status and wellbeing beyond the reach of current therapeutic modalities.

## Data Availability

Not applicable.

## Abbreviations

BD: Bipolar disorder
ATP: Adenosine triphosphate
CNS: Central nervous system
UA: Uric acid
NFAT: Nuclear factor of activated T cells
GSK-3β: Glycogen synthase kinase 3β
MDD: Major depressive disorder
CSF: Cerebrospinal fluid
NMDA: N-methyl-d-aspartate
ROS: Reactive oxygen species
NAA: N-acetyl-aspartate
BDNF: Brain-derived neurotrophic factor
RBC: Red blood cell
CVD: Cardiovascular disease
NO: Nitric oxide
TBARS: Thio barbituric acid reactive substances
LPO: Lipid peroxidase
SOD: Superoxide dismutase
ER: Endoplasmic reticulum
GWAS: Genome-wide association study
MAM: Mitochondria-associated-membrane
UPR: Unfolded protein response
SNP: Single nucleotide polymorphism
ANK3: Ankyrin-3
DISC1: Disrupted-in-schizophrenia 1
NADH: Nicotinamide adenine dinucleotide
DHEA: Dehydroepiandrosterone
PCP: Phencyclidine
VPA: Valproic acid
ASC: Apoptosis-associated speck-like protein
PAMP: Pathogen-associated molecular patterns
DAMP: Danger-associated molecular patterns
BHB: Beta-hydroxybutyrate
BMI: Body mass index
BBB: Blood–brain barrier
MBL: Mannan binding lectin
MHC: Major histocompatibility complex
PET: Positron emission tomography
NAC: N-acetylcysteine
BCAA: Branched-chain amino acid
Omega-3: Omega-3 polyunsaturated fatty acid
KYN: Kynurenine
TRP: Tryptophan
IDO: Indoleamine-pyrrole 2,3-dioxygenase
TDO: Tryptophan dioxygenase
KynA: Kynurenic acid
QA: Quinolinic acid
3HK: 3-Hydroxykynurenine
T2DM: Type-2 diabetes mellitus
LPS: Liposaccharide
AhR: Aryl hydrocarbon receptor
ECT: Electroconvulsive therapy
COX: Cyclooxygenase inhibitor
SCN: Suprachiasmatic nucleus
CCG: Central Clock genes
NAS: N-acetylserotonin
SP: Seasonal patterning
HPA: Hypothalamic–pituitary–adrenal axis
CRH: Corticotropin-releasing hormone
VTA: Ventral tegmental area
SH: Serotonergic hallucinogens
